# Identification of Functionally Interconnected Neurons Using Factor Analysis

**DOI:** 10.1155/2017/8056141

**Published:** 2017-04-16

**Authors:** Jorge H. Soletta, Fernando D. Farfán, Ana L. Albarracín, Alvaro G. Pizá, Facundo A. Lucianna, Carmelo J. Felice

**Affiliations:** ^1^Laboratorio de Medios e Interfases, Departamento de Bioingeniería (DBI), Facultad de Ciencias Exactas y Tecnología (FACET), Universidad Nacional de Tucumán (UNT), San Miguel de Tucumán, Argentina; ^2^Instituto Superior de Investigaciones Biológicas (INSIBIO), Consejo Nacional de Investigaciones Científicas y Técnicas (CONICET), San Miguel de Tucumán, Argentina

## Abstract

The advances in electrophysiological methods have allowed registering the joint activity of single neurons. Thus, studies on functional dynamics of complex-valued neural networks and its information processing mechanism have been conducted. Particularly, the methods for identifying neuronal interconnections are in increasing demand in the area of neurosciences. Here, we proposed a factor analysis to identify functional interconnections among neurons via spike trains. This method was evaluated using simulations of neural discharges from different interconnections schemes. The results have revealed that the proposed method not only allows detecting neural interconnections but will also allow detecting the presence of presynaptic neurons without the need of the recording of them.

## 1. Introduction

The microelectrodes array technology is a standard tool in the neuroscience field. This technology has allowed observing the activity of neuronal populations, establishing specific correlations between them, and revealing different strategies for processing of sensory information [1–3]. The temporal correlations among spike trains have been associated with the neural coding, stimuli discrimination, and process related to attention [4]. Furthermore, it has been suggested that such correlations are due to the anatomical and functional interconnection between neural networks of the underlying tissue [5]. Thus, the spatio/temporal knowledge of neural interconnections is currently of great interest in the study of the sensory neural code.

Processing techniques, such as cross-correlation and Granger causality, are often used to establish connections between neurons [6, 7], while other multivariate methods, such as generalized linear model (GLM), directed transfer function (DTF), or partial directed coherence (PDC), allow identifying interconnections in neuronal populations [8–10]. All these methods require that the functional activities of neurons, or neuronal groups, are electrophysiologically registered. However, it is often necessary to identify functional interconnections between neurons whose activity is known with others whose activity is unknown, and, for this, very few processing techniques can be used. It is known that the neural facilitation/inhibition of retinal ganglion responses changes when these are stimulated outside of their receptive fields [11, 12] and that this is due to interconnections between ganglion cells with presynaptic neurons [13]. This situation cannot be studied by conventional methods, since the simultaneous recording of ganglion cells and presynaptic neurons cannot be performed. Thus, a method that reveals such interconnections would be of great interest in the neurosciences area.

Here, we propose a multivariate technique (factor analysis) to detect and to establish functional interconnections between neurons. The factor analysis is a well-known statistical analysis technique; however, it was not used to detect interconnections in the sensory systems based on response of single neurons (spikes). The robustness of the proposed method was assessed through computational simulations. Our results show that factor analysis allows identifying neuronal interconnections among neurons with known response (i.e., ganglion cells), in addition to those with unknown response (i.e., presynaptic neurons).

## 2. Materials and Methods

### 2.1. Generation of Synapses Interconnection

Synaptic interactions and interconnections schemes were modeled in two ways: the first using a model of* correlated currents* and the second form is using* neural networks*.

The model of* correlated currents* consists in generating fluctuations of presynaptic currents using [14]

(1) $$\begin{array}{c} S_{i}=\mu_{i}+\sigma_{i}\left(\;\sqrt{1-p}\cdot I_{i}\left(\;t\;\right)+\sqrt{p}\cdot I_{Cj}\left(\;t\;\right)\;\right), \end{array}$$

where *μ*_*i*_ is the temporal average of the current. The second term represents fluctuations along time and it is composed of the weighted sum of two factors: *I*_*i*_ is the current of *i*th neuron, *I*_*Cj*_ is the current of presynaptic neuron *C*_*j*_, *p* (0 < *p* < 1) is the presynaptic correlation values of *I*_*i*_ and *I*_*Cj*_ currents, and *σ*_*i*_ is the variance of input current. These currents have a distribution of white noise, and in all cases these have a temporal length of 5 seconds.

**Figure 1:**
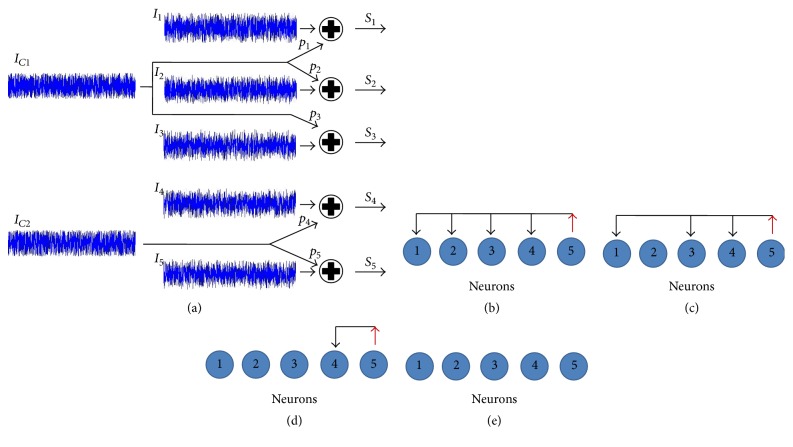
Neuronal interconnection schemes. (a) Schemes based on neuronal currents: *C*_1_ neuron synapses with *n*_1_, *n*_2_, and *n*_3_ neurons, while *C*_2_ neuron synapses with *n*_4_ and *n*_5_. (b), (c), (d), and (e) are different neuronal interconnection schemes. The black arrows indicate the direction of presynaptic current, while the red arrows indicate the neuron that generates the presynaptic current and whose activity is independent of the others. All other neurons to fire independently (without arrows).

The simulated neuronal interconnections are shown in Figure 1(a). The presynaptic current *I*_*C*1_ has connections with *n*_1_, *n*_2_, and *n*_3_ neurons, and it is weighted by *p*_1_, *p*_2_, and *p*_3_, correlation values, respectively. Likewise, *I*_*C*2_ current has connections with *n*_4_ and *n*_5_ neurons.

The correlated current values, *S*_*i*_, were inputs of Leaky Integrate-and-Fire (LIF) Neuron Model which was used as spike generator. The parameters used in the model were as follows: membrane time constant (tau) tau = 10 ms, membrane potential threshold = −55 mV, resting potential = −70 mV, and reset value = −75 mV. Thus, the spike trains from *n*_1_, *n*_2_, *n*_3_, *n*_4_, and *n*_5_ neurons were obtained. Then, the spike count within a window of 50 ms was determined.

Because the model of correlated current has very strong modeling assumptions about the structure of correlations and generation of postsynaptic currents, it was decided additionally to use a model based on neural networks.

**Figure 2:**
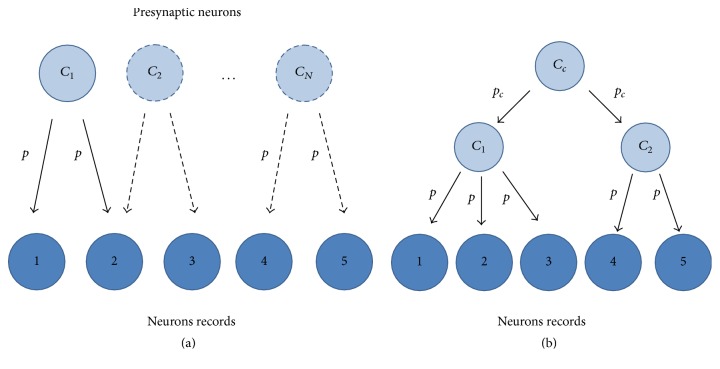
Neuronal interconnection schemes. (a) *C*_1_, *C*_2_,…, *C*_*N*_ are presynaptic neurons. The solid arrows indicate a fixed connection, while dashed arrows indicate the probabilistic connections. These last connections are given between a presynaptic neuron and a pair of recorded neurons. (b) Scheme neuronal interconnections with two layers of presynaptic neurons. Discharges of *C*_1_ and *C*_2_ neurons are correlated by the discharge of neuron *C*_*c*_.

Thus, the second method used to model the neural interconnections consists in using feed-forward network [15]. Neural baseline activity is given by uncorrelated homogeneous Poisson processes. For different simulations, the rate parameter was varied (*λ* = 1/10, 1/25, and 1/50) corresponding to level of spontaneous spiking activity. The synaptic weights (*w*_*i*_) took different values according to different situations analyzed. In the simulations with the interconnections of Figures 1(b), 1(c), 1(d), and 1(e), the synaptic weights took deterministic values, while in the scheme of Figure 2(a) the weights took values with uniform distribution (parameters [0–0.3] and [0–0.8]). The nonlinear combination of all inputs was deterministically performed through logistic sigmoid function (activation function):

(2) $$\begin{array}{c} f\left(\;x\;\right)=\frac{1}{1+e^{-a\left(x-c\right)}}, \end{array}$$

where *a* = 1 and *c* = 0.8 [16].

### 2.2. Factor Analysis

The factor analysis (FA) is based on the following model [17]:

(3) $$\begin{array}{c} x=a+\Delta f+u, \end{array}$$

where *x* is a vector of *n* × 1 which contain the spike count values of all neurons, *a* is a mean vector (*n* × 1), *f* is a vector of *m* × 1 which contain the unobservable factors—spike count values of presynaptic neurons—Δ is a loading matrix of *n* × *m*, and *u* is a vector of *n* × 1 with unobservable perturbations, which has a multivariate normal distribution of order *n*—*N*_*n*_(0, *ψ*), with the components of mean vector 0 being equal to zero, and the variance matrix *ψ* is equal to identity. Thus, *x* vector has a multivariate normal distribution, *N*_*n*_(*a*, *V*).

The amount of factors is related to the amount of presynaptic neurons; that is, if two factors are considered in the model, then two presynaptic neurons are considered in the analysis. Δ will be related to *p*_*i*_ correlation values in the interconnection model proposed, and it will have five rows (one for each recorded neuron) and two columns (one for each presynaptic neuron). Finally, the maximum likelihood method is used to estimate the loading matrix Δ. This method consists in finding Δ and *ψ* values which maximize the following function:

(4) LΔ,ψ=−r2log⁡Δ·Δ′+ψ+tr⁡SΔ·Δ′+ψ−1,

where *r* is the amount of time intervals where the spike count was done, *S* is the sample covariance matrix, and tr(*x*) indicates the trace of *x* matrix. The parameters that maximize the *L*(Δ, *ψ*) convex function are obtained through their corresponding partial derivatives (Statistical toolbox, MATLAB).

### 2.3. Metrics

The matrix 2-norm, *N*_*d*_, of the difference between calculated loading matrix and optimum loading matrix, was proposed to quantify the results obtained (see (4)). *N*_*d*_ belongs to the interval [0, inf) and can be interpreted as the distance, or discrepancy, between the loading matrix and optimum loading matrix. *N*_*d*_ = 0 when both matrices are identical. The optimum loading matrix, Δ_0_, reflects the preestablished interconnections; in the case of Figure 1(a) it is as follows:

(5) Δ0=1010100101,Nd=normΔ−Δ0.

### 2.4. Identification of Presynaptic Interconnections

The synaptic interconnections were determined by comparing the elements of loading matrix. These comparisons were performed in two steps: by rows and then by columns.

First, the position of the element with greatest value is identified through analysis of each of rows of the loading matrix. Thus, the presynaptic neuron which is connected to recorded neuron is determined. The proposed method will not be able establish any connection, if the value of any element of a row is not significantly higher compared to the others.

The previous procedure is applied to all recorded neurons. Then, each of the columns of the loading matrix is similarly analyzed. If there is one or more elements whose values are significantly higher compared to the remaining elements (in the same column), then, such positions (recorded neurons) are connected to the same presynaptic neuron.

Considering the above, a neural interconnection is determined if the following conditions are met:

- The position of the element with the highest loading value of each row coincides with the elements position of “1” value of the corresponding rows of Δ_0_ matrix.
- The loading values must be significantly higher compared to the remaining elements of the same column.

Two values are considered significantly different if their difference exceeds a preestablished threshold. This threshold was established empirically in 0.03 for proposed case studies. For future implementations, it should be taken into account that a high threshold will allow establishing stronger connections, while a low threshold will allow establishing weaker connections. On the last case, the result could contain a greater number of false positives.

## 3. Results

### 3.1. Influence of Weightings

The influence of *p*_*i*_ neuronal correlation values on the identification of interconnections was analyzed through simulations in which *p*_*i*_ value varies while *μ* and *σ* remain constant (*μ* = 3 and *σ* = 1.5). Then, the identification of interconnections was quantitatively assessed through *N*_*d*_.

**Figure 3:**
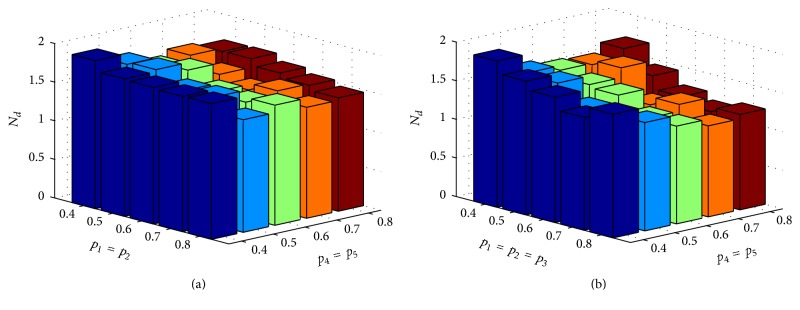
Quantification of the difference between calculated loading matrix and optimum loading matrix for interconnection scheme of Figure 1(a). (a) Mean values of *N*_*d*_ for different weighting values. 40 simulations were performed for each situation. The weighting value was *p*_3_ = 0 for all cases. (b) Mean values of *N*_*d*_ for different weighting values. 40 simulations were performed for each situation and with *σ* = 1.5 and *μ* = 3.

Figure 3(a) shows *N*_*d*_ values for interconnections scheme of Figure 1(a) and considering *p*_3_ = 0. It is possible to note that *N*_*d*_ values decrease as *p*_1_, *p*_2_, *p*_4_, and *p*_5_ values increase. This trend is most noticeable when *p*_1_ = *p*_2_ = *p*_3_, Figure 3(b).

### 3.2. Influence of the Samples Number

**Figure 4:**
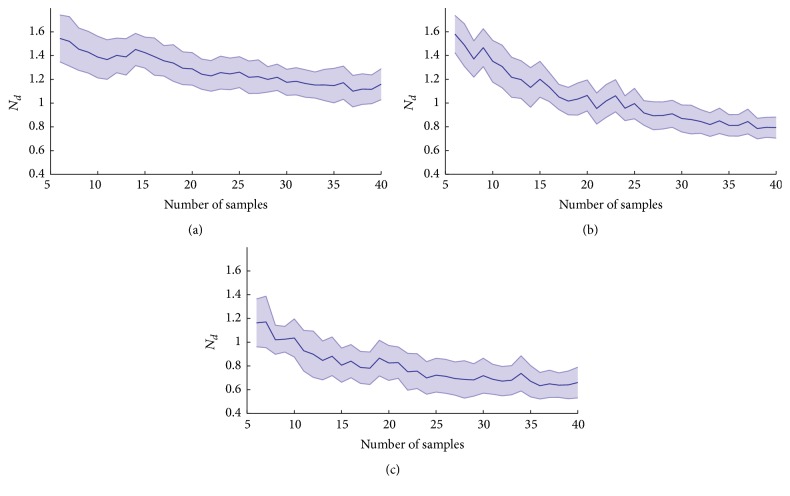
Quantification of the difference between the calculated loading matrix and optimum loading matrix (*N*_*d*_ values) as a function of the samples number used (*r* values, i.e., the samples number or the amount of time intervals used) for interconnections scheme of Figure 1(a). (a) The weighting values were equal to *p* = 0.4 for all connections. (b) *p* = 0.6 for all connections. (c) *p* = 0.8 for all connections. The line indicates the mean while the lightest area is the standard deviation. Forty repetitions were realized in all cases.

*N*_*d*_ values for the neuronal interconnections of Figure 1(a), versus the samples number (*r*), is shown in Figure 4. It is observed that *N*_*d*_ values decrease and converge to a value when the samples number is increased. The convergence values are 1.1, 0.8, and 0.6 for *p* values equaling 0.4, 0.6, and 0.8, respectively.

### 3.3. Identification of Interconnections from Different Loading Matrices

Different interconnections schemes were proposed for assessing the factor analysis procedure (Figures 1(b)–1(e)). Spike trains from five neurons were recorded in all cases. The average loading matrices for interconnection scheme of Figure 1(b) are shown in Tables 1(a) and 1(b). It is important to clarify that the weighting or correlation values should be varied independently to make a more intensive analysis of the proposed method. However, as a first approximation and to simplify the results, the case where all weights are equal to each other has been considered. For the two models of interconnections (Tables 1(a) and 1(b)) it is observed that the values of the first column are higher than those of second column for different weighting values indicating the existence of a presynaptic neuron. Likewise, the loading matrix reveals that *n*_5_ is the presynaptic neuron (highest values) when comparing the elements of the first columns. This difference between the values of the first column is greater in the model correlated currents (Table 1(a)).

Similarly, Table 2 shows the average loading matrices obtained for the interconnection scheme of Figure 1(c). Spike trains from five neurons were recorded for all the experiments, while the values of *n*_5_ neuron are the highest when comparing the elements of the first columns. This particularity indicates that *n*_2_ neuron would not be connected to other neurons and that it would fire independently (italic font values) and also that *n*_5_ is presynaptic to the other neurons (bold font values).

The average loading matrices obtained for the interconnection scheme of Figure 1(d) are shown in Table 3. It is observed that the values of *n*_4_ and *n*_5_ neurons (first column of matrices) are similar to each other and higher values than those belonging to other neurons. The difference of values between the first and second column increases with *p*_4_ weighting. For this situation, it is not possible to determine the direction of the neural connection because of the similarity between the values of *n*_4_ and *n*_5_ neurons. Thus, the loading matrix only would indicate the existence of a common presynaptic neuron.

In Tables 4(a) and 4(b) (loading matrix of Figure 1(e)) it is observed that the values of both columns are similar for all neurons. This indicates that the method cannot identify a pattern of neuronal interconnection which agrees with the scheme of Figure 1(e).

### 3.4. Influence of the Number of Presynaptic Neurons

The influence of presynaptic neurons number in the calculation of the “loading matrix” was analyzed. For this, neural network model was used, and scheme used interconnections shown in Figure 2(a). In this case only a presynaptic neuron connected to neurons 1 and 2 with *p* = 0.8 value, while *p* values for the other *N* − 1 connections between presynaptic neurons and neurons registration set at random and with different values of *λ*. In Table 5 the theoretical loading matrix for the interconnection scheme of Figure 2(a) is observed. In Tables 6(a), 6(c), and 6(e), it is observed that by increasing the number of presynaptic neurons the difference between the maximum and minimum values tends to decrease but still identifying interconnections is correct.

In Tables 6(d), 6(b), and 6(f), it is observed that by increasing the number of presynaptic neurons values in the first column of the loading matrix take similar values to each other. This causes the correct identification of the presynaptic neuron interconnections with greater weight (*p* = 0.8) being limited by two factors, first by the number of presynaptic neurons and second by the value of lambda. The correct identifications can be made until the number of presynaptic neurons is equal to *N* = 50, 65, and 80 for *λ* values 1/10, 1/25, and 1/50, respectively.

### 3.5. Influence of Correlation between Trains Spikes Presynaptic Neurons

The simulations above meet the condition that the discharges of presynaptic neurons are independent. Now we will study the robustness of FA method when trains spikes of presynaptic neurons are correlated.

The neuronal interconnections for scheme of Figure 2(b) were established by using the correlated current model. In this scheme, the discharge of *C*_1_ and *C*_2_ presynaptic neurons is correlated by the discharges of *C*_*c*_ neuron. The loading matrices for this situation were obtained applying the FA method to 1, 2, 3, 4, and 5 recorded neurons (Table 7); in Table 8 the theoretical loading matrix for the interconnection scheme is observed. It is observed that, for low correlation values (*p*_*c*_ = 0.2), FA can correctly identify the structure of neuronal interconnections. For greater correlation values (*p*_*c*_ ≥ 0.4) FA presents problems to correctly detect the structure of interconnections.

## 4. Discussion and Conclusion

The FA is a multivariate statistical method that has traditionally been applied in the psychology areas and recently in neuroscience areas. Thus, for example, specific functional interconnections between neuronal groups have been established through the factor analysis [18]. Yu et al. propose using an extension of FA (Gaussian-process factor analysis, GPFA) to detect and model modulatory or underlying neural processes that modify the response of neuronal populations over time [19, 20].

The correct identification of connections between neurons and/or neuronal groups is a problem of interest in neuroscience. In this aspect many techniques to identify functional interconnections have been proposed, but most are limited to the interconnection of neuronal groups with a large number of neurons [8–10, 21].

New advances in electrophysiological methods have allowed registering the joint activity of single neurons, so that a more specific functional analysis could be conducted. Thus, the methods for identifying neuronal interconnections via spike trains are a growing demand in the area of neurosciences. Thus, for example, the influence of the activity of interneurons, or presynaptic neurons (no recorded), on the activity recorded from other neurons, could be of great interest. Echtermeyer et al. have proposed a technique capable of detecting the presence of interneurons whose activity was unknown but interconnected with other neurons whose activity was known. However, it was not capable of detecting interconnections between neurons whose activities are known with presynaptic neurons whose activities are unknown [22].

In this study we have proposed a factor analysis to identify functional interconnections among neurons by using spike trains. Factor analysis is a statistical technique, and to be applied it is necessary to verify the fulfillment of its hypotheses. In this aspect the robustness of the technique applied to neural responses was validated by comparing the results obtained with ideal results, particularly for this study comparing interconnections obtained with FA and imposed by the model.

In order that the FA can be used to identify neural interconnections the following assumptions must be met:

- Trains presynaptic neurons spikes should be independent or have a low correlation between them.
- Recorded spikes trains should not have temporal correlation.

It is not possible to verify a priori the independence between spike trains from presynaptic neurons by using real recordings. Therefore the assumptions necessary to validate the results obtained with the proposed method must be corroborated through the anatomical/functional knowledge of the analyzed system. For example, it is known that the amacrine cells of the retina, which are presynaptic of the ganglion cells, fire independently. Thus, the proposed method could be used in retina.

Our results, based on computational simulations, have revealed that the proposed method not only allows detecting neural interconnections but will also allow detecting the presence of presynaptic neurons without the need of the recording of them.

The neuronal interconnection schemes proposed in this study were chosen because of their similarity to those commonly found in the nervous system. Thus, for example, the neuronal interconnections of Figure 1(a) are biologically plausible in the human retina and are given by ganglion and amacrine cells [13], whereas those proposed in Figures 1(b), 1(c) and 1(d) could be found in cortical areas [23].

Although the results found are based on computational simulations, FA could be applied to real neural recordings. For this, neuronal recordings must meet specific conditions (listed above) such as those related to temporal correlation.

In the simulations we have used two methods to generate trains of spikes and interconnections. The results have shown that the efficiency of FA in identifying interconnections does not depend on the method used in generating trains of spikes but rather depends on the probabilistic structure (whether or not there is correlation). In addition the results obtained revealed that there are two factors that influence the efficiency of the method, first the number of presynaptic neurons and secondly the weight of synapses.

## Figures and Tables

**(a):** 

*p*_1_ = *p*_2_ = *p*_3_ = *p*_4_									
0.4		0.5		0.6		0.7		0.8	
0.5446	0.2068	0.6618	0.2387	0.7726	0.1821	0.8427	0.2088	0.9175	0.1453
0.5431	0.2159	0.6123	0.2333	0.7469	0.2302	0.8569	0.1600	0.9118	0.1188
0.4837	0.2708	0.6568	0.2509	0.7567	0.2330	0.8521	0.1740	0.9233	0.0825
0.4943	0.2389	0.6198	0.2117	0.7362	0.2365	0.8537	0.1446	0.9229	0.1163
**0.7640**	**0.3233**	**0.8219**	**0.3141**	**0.8627**	**0.2745**	**0.9069**	**0.2196**	**0.9457**	**0.1314**

**(b):** 

*w*_1_ = *w*_2_ = *w*_3_ = *w*_4_									
0.4		0.5		0.6		0.7		0.8	
0.6438	0.1870	0.7084	0.1869	0.7460	0.1768	0.7677	0.1855	0.8181	0.1935
0.5450	0.2574	0.6003	0.3214	0.7135	0.2170	0.7894	0.1752	0.8138	0.1939
0.5484	0.2319	0.6549	0.2543	0.7341	0.2036	0.7838	0.1994	0.8377	0.1560
0.5542	0.2740	0.6756	0.2238	0.7303	0.1923	0.7864	0.1832	0.8346	0.1446
**0.6960**	**0.4122**	**0.7356**	**0.4024**	**0.7946**	**0.2709**	**0.8305**	**0.2164**	**0.8582**	**0.1835**

**(c):** 

Δ_0_	
1	0
1	0
1	0
1	0
**1**	0

**(a):** 

*p*_1_ = *p*_3_ = *p*_4_									
0.4		0.5		0.6		0.7		0.8	
0.5664	0.1916	0.6303	0.2916	0.7394	0.2184	0.8158	0.2082	0.8947	0.1402
*0.0257*	*0.1801*	*0.0163*	*0.2127*	*0.0411*	*0.1778*	*0.0218*	*0.2919*	*0.0342*	*0.2696*
0.4897	0.2595	0.6704	0.1514	0.7506	0.1856	0.8321	0.1741	0.8931	0.1447
0.5055	0.2256	0.6636	0.1495	0.7559	0.2125	0.8360	0.1802	0.8922	0.1545
**0.6901**	**0.2901**	**0.8155**	**0.2686**	**0.8637**	**0.2762**	**0.8906**	**0.2148**	**0.9192**	**0.1665**

**(b):** 

*w*_1_ = *w*_3_ = *w*_4_									
0.4		0.5		0.6		0.7		0.8	
0.6115	0.0726	0.6992	0.0554	0.7659	0.0530	0.8071	0.0543	0.8434	0.0590
*−0.0031*	*0.4820*	*0.0009*	*0.5207*	*−0.0017*	*0.4615*	*0.0004*	*0.4860*	*0.0004*	*0.4930*
0.6147	0.0688	0.6952	0.0806	0.7686	0.0622	0.8175	0.0400	0.8518	0.0387
0.6179	0.0908	0.7002	0.0551	0.7582	0.0775	0.8122	0.0411	0.8549	0.0435
**0.8663**	**0.0692**	**0.8673**	**0.0736**	**0.8785**	**0.0630**	**0.8852**	**0.0446**	**0.8865**	**0.0470**

**(c):** 

Δ_0_	
1	0
0	0
1	0
1	0
**1**	0

**(a):** 

*p*_4_									
0.4		0.5		0.6		0.7		0.8	
*0.0991*	*0.1425*	*0.0891*	*0.1396*	*0.1199*	*0.1348*	*0.1187*	*0.1162*	*0.0906*	*0.1252*
*0.0908*	*0.1416*	*0.1202*	*0.1888*	*0.0411*	*0.1881*	*0.0849*	*0.1668*	*0.0252*	*0.1818*
*0.1409*	*0.1768*	*0.1194*	*0.1703*	*0.0384*	*0.1497*	*0.0736*	*0.2176*	*0.0652*	*0.1699*
0.4898	0.2621	0.6305	0.2257	0.6563	0.2866	0.6827	0.2726	0.7660	0.2773
**0.4836**	**0.3162**	**0.6131**	**0.2428**	**0.6634**	**0.2752**	**0.6945**	**0.2844**	**0.7548**	**0.2783**

**(b):** 

*w*_4_									
0.4		0.5		0.6		0.7		0.8	
*0.0177*	*0.1103*	*0.0002*	*0.1450*	*0.0128*	*0.1025*	*0.0021*	*0.1213*	*0.0080*	*0.1557*
*0.0211*	*0.0886*	−*0.0065*	*0.1089*	−*0.0205*	*0.1207*	*0.0013*	*0.0988*	−*0.0386*	*0.1895*
*0.0021*	*0.1352*	−*0.0087*	*0.1229*	−*0.0046*	*0.1646*	−*0.0298*	*0.1399*	*0.0139*	*0.0893*
0.7149	0.1680	0.7720	0.1635	0.7584	0.1827	0.7799	0.1899	0.8223	0.1570
**0.6886**	**0.1702**	**0.7427**	**0.1590**	**0.8068**	**0.1620**	**0.8133**	**0.2037**	**0.8303**	**0.1491**

**(c):** 

Δ_0_	
0	0
0	0
0	0
1	0
**1**	0

**(a):** 

A		B	
0.2567	0.1889	0.1683	0.0857
0.2281	0.1849	0.1841	0.0724
0.1705	0.1684	0.1569	0.1192
0.2170	0.1788	0.1843	0.1197
0.1895	0.1844	0.1657	0.0819

**(b):** 

Δ_0_	
0	0
0	0
0	0
0	0
0	0

**Table 5:** Theoretical loading matrix for the interconnection scheme of Figure 2(a).

Δ_0_	
1	0
1	0
0	0
0	0
0	0

**(a):** 

*w*_*i*_ ~ *U*[0–0.3]													
*N*													
5		20		35		50		65		80		95	
**0.7779**	**0.1596**	**0.7299**	**0.1983**	**0.7202**	**0.1748**	**0.6540**	**0.1726**	**0.6563**	**0.1902**	**0.5419**	**0.2154**	**0.5848**	**0.1786**
**0.7938**	**0.1792**	**0.7429**	**0.1882**	**0.6838**	**0.1967**	**0.6847**	**0.1785**	**0.6192**	**0.1805**	**0.6071**	**0.1890**	**0.5517**	**0.1861**
0.0307	0.1540	0.1150	0.1404	0.1598	0.1994	0.2034	0.2212	0.1907	0.2671	0.2528	0.2599	0.2402	0.2870
0.0630	0.1233	0.1268	0.1862	0.1432	0.2329	0.1937	0.2121	0.1766	0.2737	0.2232	0.2401	0.2239	0.2912
0.0586	0.1356	0.0998	0.1676	0.1558	0.1987	0.1508	0.2407	0.2070	0.2655	0.2282	0.2642	0.2476	0.2777

**(b):** 

*w*_*i*_ ~ *U*[0–0.8]													
*N*													
5		20		35		50		65		80		95	
**0.6982**	**0.1678**	**0.4941**	**0.2160**	**0.4485**	**0.2618**	**0.3989**	**0.3010**	*0.3616*	*0.2963*	*0.3825*	*0.2585*	*0.3598*	*0.2838*
**0.6921**	**0.1681**	**0.5088**	**0.2602**	**0.4535**	**0.2817**	**0.4179**	**0.2445**	*0.3905*	*0.2699*	*0.3762*	*0.2903*	*0.3606*	*0.2893*
0.1768	0.1196	0.3182	0.1746	0.3765	0.1667	0.3324	0.2689	0.3425	0.2412	0.3217	0.2595	0.3786	0.2530
0.2076	0.1492	0.3365	0.1716	0.3306	0.1912	0.3416	0.2607	0.3797	0.2277	0.3421	0.2591	0.3476	0.2430
0.1646	0.1214	0.3410	0.1471	0.3268	0.2483	0.3357	0.2591	0.3914	0.2421	0.4013	0.2372	0.3609	0.2636

**(c):** 

*w*_*i*_ ~ *U*[0–0.3]													
*N*													
5		20		35		50		65		80		95	
**0.7670**	**0.2023**	**0.7204**	**0.2098**	**0.6968**	**0.1470**	**0.6680**	**0.1753**	**0.6530**	**0.1995**	**0.5653**	**0.2034**	**0.5419**	**0.2155**
**0.8035**	**0.1910**	**0.7323**	**0.2285**	**0.7401**	**0.1504**	**0.6549**	**0.1893**	**0.6246**	**0.1614**	**0.6239**	**0.1958**	**0.6027**	**0.1974**
0.0326	0.1232	0.1252	0.1612	0.1354	0.2676	0.1858	0.2101	0.2074	0.2580	0.2272	0.2668	0.2413	0.2682
0.0484	0.1296	0.1580	0.0948	0.1290	0.2413	0.1694	0.2529	0.1759	0.2604	0.2248	0.2673	0.2161	0.2855
0.0092	0.1467	0.1421	0.1219	0.1525	0.2517	0.1789	0.2427	0.1926	0.2528	0.1941	0.2872	0.2178	0.2864

**(d):** 

*w*_*i*_ ~ *U*[0–0.8]													
*N*													
5		20		35		50		65		80		95	
**0.6716**	**0.2985**	**0.5202**	**0.2066**	**0.4485**	**0.2552**	**0.4248**	**0.2359**	**0.4223**	**0.2723**	*0.3872*	*0.2616*	*0.4204*	*0.2659*
**0.6760**	**0.2754**	**0.5327**	**0.2183**	**0.4445**	**0.2749**	**0.3838**	**0.2581**	**0.4068**	**0.2642**	*0.3781*	*0.2668*	*0.3676*	*0.2873*
0.2218	0.0296	0.3302	0.1424	0.3377	0.2303	0.3594	0.2363	0.3459	0.2788	0.3365	0.2680	0.3846	0.2310
0.2157	0.0256	0.3329	0.1433	0.3403	0.2058	0.3455	0.2620	0.3745	0.2079	0.3613	0.2335	0.3321	0.2650
0.1783	0.0592	0.3473	0.1678	0.3543	0.1928	0.3523	0.2753	0.2936	0.2653	0.3879	0.2419	0.3346	0.2677

**(e):** 

*w*_*i*_ ~ *U*[0–0.3]													
*N*													
5		20		35		50		65		80		95	
**0.7695**	**0.2005**	**0.7171**	**0.2197**	**0.6964**	**0.1806**	**0.6534**	**0.2037**	**0.6770**	**0.1593**	**0.5918**	**0.1889**	**0.5947**	**0.1995**
**0.7955**	**0.1910**	**0.7411**	**0.2220**	**0.7267**	**0.1840**	**0.6376**	**0.1902**	**0.6489**	**0.1674**	**0.6145**	**0.1758**	**0.5526**	**0.2018**
0.0224	0.1368	0.1281	0.1308	0.1526	0.2038	0.2029	0.2308	0.1569	0.2732	0.2358	0.2607	0.2134	0.3068
0.0627	0.0887	0.1499	0.0980	0.1624	0.2018	0.1996	0.2070	0.1814	0.2778	0.1896	0.2789	0.1965	0.2827
0.0390	0.1392	0.1373	0.1527	0.1660	0.1754	0.2037	0.2011	0.1881	0.2762	0.1971	0.2845	0.2661	0.2286

**(f):** 

*w*_*i*_ ~ *U*[0–0.8]													
*N*													
5		20		35		50		65		80		95	
**0.6770**	**0.2134**	**0.5146**	**0.1900**	**0.4295**	**0.2729**	**0.4231**	**0.2529**	**0.3905**	**0.2776**	**0.3709**	**0.2746**	*0.3788*	*0.2737*
**0.6921**	**0.2181**	**0.5260**	**0.2292**	**0.4421**	**0.2741**	**0.4141**	**0.3014**	**0.3923**	**0.2579**	**0.4017**	**0.3031**	*0.3502*	*0.3137*
0.1844	0.0757	0.3626	0.1591	0.3517	0.1709	0.3205	0.2360	0.3650	0.2447	0.3548	0.2492	0.3591	0.2522
0.2169	0.0395	0.3250	0.1595	0.3569	0.2014	0.3490	0.2394	0.3656	0.2211	0.3665	0.2603	0.3396	0.2625
0.1845	0.0934	0.3108	0.1587	0.3678	0.2014	0.3646	0.2135	0.3420	0.2606	0.3396	0.2717	0.4024	0.2383

**Table 7:** Average loading matrices obtained for the interconnection scheme of Figure 2(b) Correlation coefficient *p* = 0.8. Two Hundred simulations were realized for each situation. They are highlighted in bold font for situations where the identification of the connections was correct and incorrect situations for italic font.

*p*_*c*_ = 0.2		*p*_*c*_ = 0.4		*p*_*c*_ = 0.6		*p*_*c*_ = 0.8	
**0.7512**	0.2870	**0.7996**	0.3363	*0.8099*	0.3835	*0.9425*	0.1459
**0.7589**	0.2844	**0.7924**	0.3617	*0.8102*	0.3884	*0.9466*	0.1392
**0.7786**	0.2904	**0.7834**	0.3528	*0.8210*	0.3706	*0.9385*	0.1432
0.0996	**0.5231**	*0.5032*	0.2033	*0.8670*	−0.0749	*0.9361*	0.0328
0.0796	**0.5319**	*0.5226*	0.1739	*0.8498*	−0.0711	*0.9497*	0.0316

**Table 8:** Theoretical loading matrix for the interconnection scheme of Figure 2(b).

Δ_0_	
1	0
1	0
1	0
0	1
0	1

## References

[1] Stopfer M., Bhagavan S., Smith B. H., Laurent G. (1997). Impaired odour discrimination on desynchronization of odour-encoding neural assemblies. *Nature*.

[2] Alonso J.-M., Usrey W. M., Reid R. C. (1996). Precisely correlated firing in cells of the lateral geniculate nucleus. *Nature*.

[3] Shlens J., Field G. D., Gauthier J. L. (2006). The structure of multi-neuron firing patterns in primate retina. *Journal of Neuroscience*.

[4] Christopher Decharms R., Merzenich M. M. (1996). Primary cortical representation of sounds by the coordination of action-potential timing. *Nature*.

[5] Diekman C., Dasgupta K., Nair V., Unnikrishnan K. P. (2014). Discovering functional neuronal connectivity from serial patterns in spike train data. *Neural Computation*.

[6] Hesse W., Möller E., Arnold M., Schack B. (2003). The use of time-variant EEG Granger causality for inspecting directed interdependencies of neural assemblies. *Journal of Neuroscience Methods*.

[7] Perkel D. H., Gerstein G. L., Moore G. P. (1967). Neuronal spike trains and stochastic point processes. II. Simultaneous spike trains. *Biophysical Journal*.

[8] Kaminski M. J., Blinowska K. J. (1991). A new method of the description of the information flow in the brain structures. *Biological Cybernetics*.

[9] Pillow J. W., Shlens J., Paninski L. (2008). Spatio-temporal correlations and visual signalling in a complete neuronal population. *Nature*.

[10] Takahashi D. Y., Baccal L. A., Sameshima K. (2007). Connectivity inference between neural structures via partial directed coherence. *Journal of Applied Statistics*.

[11] Ölveczky B. P., Baccus S. A., Meister M. (2003). Segregation of object and background motion in the retina. *Nature*.

[12] McIlwain J. T. (1964). Receptive fields of optic tract axons and lateral geniculate cells: peripheral extent and barbiturate sensitivity. *Journal of Neurophysiology*.

[13] Geffen M. N., de Vries S. E. J., Meister M. (2007). Retinal ganglion cells can rapidly change polarity from off to on. *PLoS Biology*.

[14] De La Rocha J., Doiron B., Shea-Brown E., Josić K., Reyes A. (2007). Correlation between neural spike trains increases with firing rate. *Nature*.

[15] Dayan P., Abbott L. F. (2005). *Theoretical Neuroscience: Computational and Mathematical Modeling of Neural Systems*.

[16] Warland D. K., Reinagel P., Meister M. (1997). Decoding visual information from a population of retinal ganglion cells. *Journal of Neurophysiology*.

[17] Peña D. (2002). *Análisis de Datos Multivariantes*.

[18] Manning J. R., Ranganath R., Norman K. A., Blei D. M. (2014). Topographic factor analysis: a bayesian model for inferring brain networks from neural data. *PLoS ONE*.

[19] Yu B. M., Cunningham J. P., Santhanam G., Ryu S. I., Shenoy K. V., Sahani M. (2009). Gaussian-process factor analysis for low-dimensional single-trial analysis of neural population activity. *Journal of Neurophysiology*.

[20] Macke J. H., Buesing L., Cunningham J. P., Yu B. M., Shenoy K. V., Sahani M. (2011). Empirical models of spiking in neural populations. *Advances in Neural Information Processing Systems 24*.

[21] Wang X.-J., Buzsáki G. (1996). Gamma oscillation by synaptic inhibition in a hippocampal interneuronal network model. *The Journal of Neuroscience*.

[22] Echtermeyer C., Smulders T. V., Smith V. A. (2010). Causal pattern recovery from neural spike train data using the Snap Shot Score. *Journal of Computational Neuroscience*.

[23] Klyachko V. A., Stevens C. F. (2003). Connectivity optimization and the positioning of cortical areas. *Proceedings of the National Academy of Sciences of the United States of America*.

